# Supplementary material for: Buffaloed in Brandenburg: Germany’s first brush with foot-and-mouth disease after four decades of freedom
Source: Sci Adv. 2026 Oct 9;12(41):eaeh6459. doi: 10.1126/sciadv.aeh6459 (PMC13651877; doi:10.1126/sciadv.aeh6459)
Supplement: Supplementary file 1 [file sciadv.aeh6459_sm.pdf]

Science Advances
AAAS

Supplementary Materials for

Buffaloed in Brandenburg: Germany's first brush with foot-and-mouth disease after four decades of freedom

Michael Eschbaumer *et al.*

Corresponding author: Martin Beer, martin.beer@fli.de

Sci. Adv. **12**, eaeh6459 (2026)
DOI: 10.1126/sciadv.aeh6459

This PDF file includes:

Figs. S1 to S5
Tables S1 to S6

Supplemental Figures

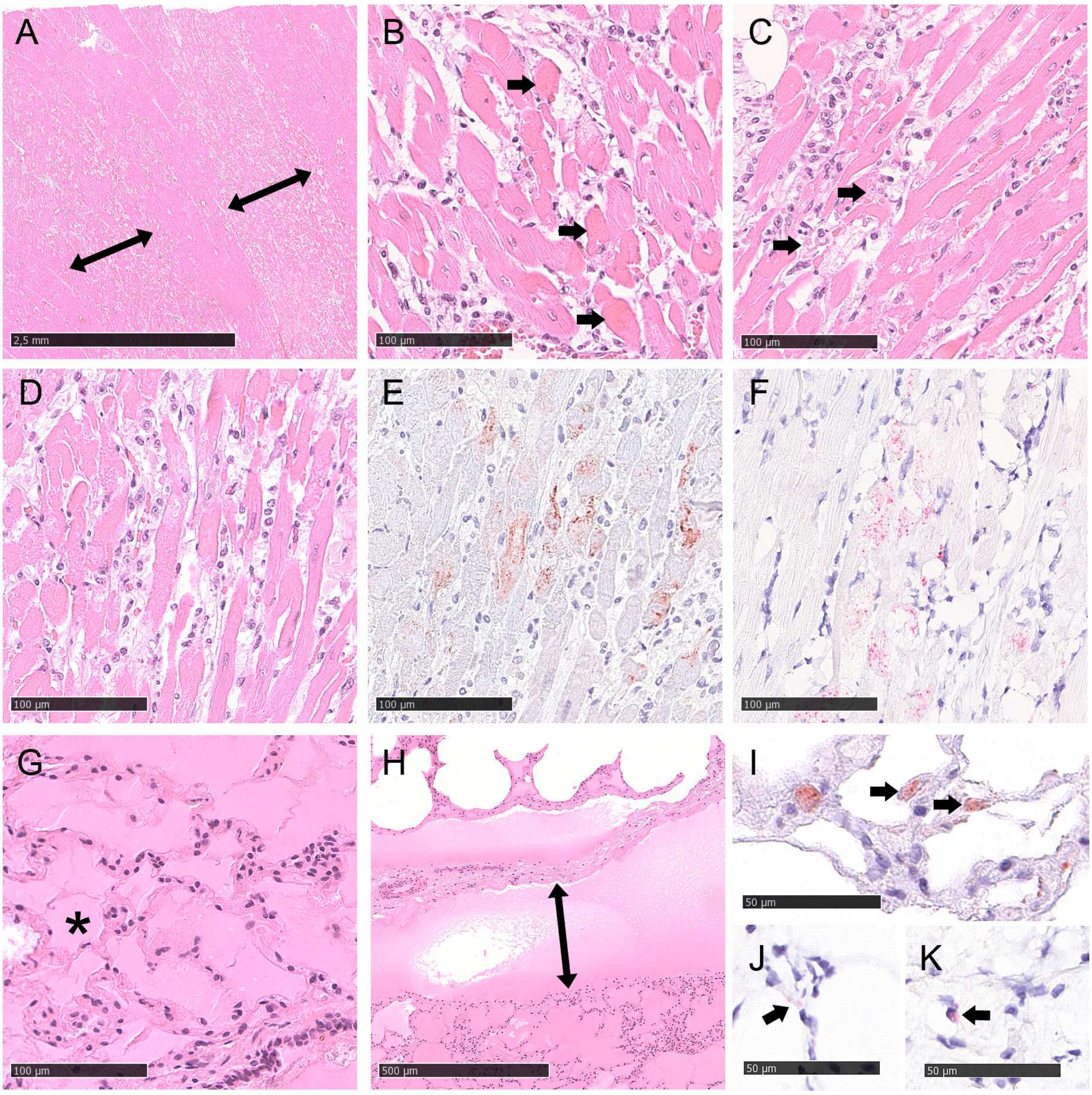

Supplemental Figure S1: Histological findings. Histopathology of heart (A-F) and lung (G-K) of buffalo 334 with detection of FMDV antigen (E, I) and RNA (F, J-K). (A) Histologically, similar to the macroscopic picture, there is streaky brightening (↕) of the myocardium. Haematoxylin and eosin (H&E) staining, bar = 2.5 mm. (B) Cardiomyocytes show either hyaline degeneration (→) with glassy, homogeneous cytoplasm, or (C) acute necrosis (→) and appear as cellular debris. Immune cell infiltrates comprise mainly lymphocytes and macrophages and are located between affected cells H&E, bar = 100 µm. (D-E) Cardiomyocytes affected and shown with H&E in (D) exhibit intralesional FMDV antigen as shown by immunohistochemistry (IHC) in (E) using 3-amino-9-ethylcarbazole (AEC) for red-brown chromogen labelling and haematoxylin counterstain and FMDV genome in (F), as shown by RNA in situ hybridization (RNA-ISH) using

fast red for labelling and a haematoxylin counterstain, bar = 100 µm. (G) Alveolar and (H) interstitial oedema with presence of eosinophilic, proteinaceous material within the alveoli (*) and expanding the interstitial (↕) space, H&E, bar (G) = 100 µm and (H) = 500 µm. (I) FMDV antigen detection in the cytoplasm of the pulmonary epithelium, IHC, AEC chromogen labelling, haematoxylin counterstain, bar = 50 µm. (J-K) FMDV genome detection in the cytoplasm of very few pulmonary epithelial cells, morphologically consistent with (J) type I and (K) type II pneumocytes. RNA-ISH, fast red labelling, haematoxylin counterstain, bar = 50 µm.

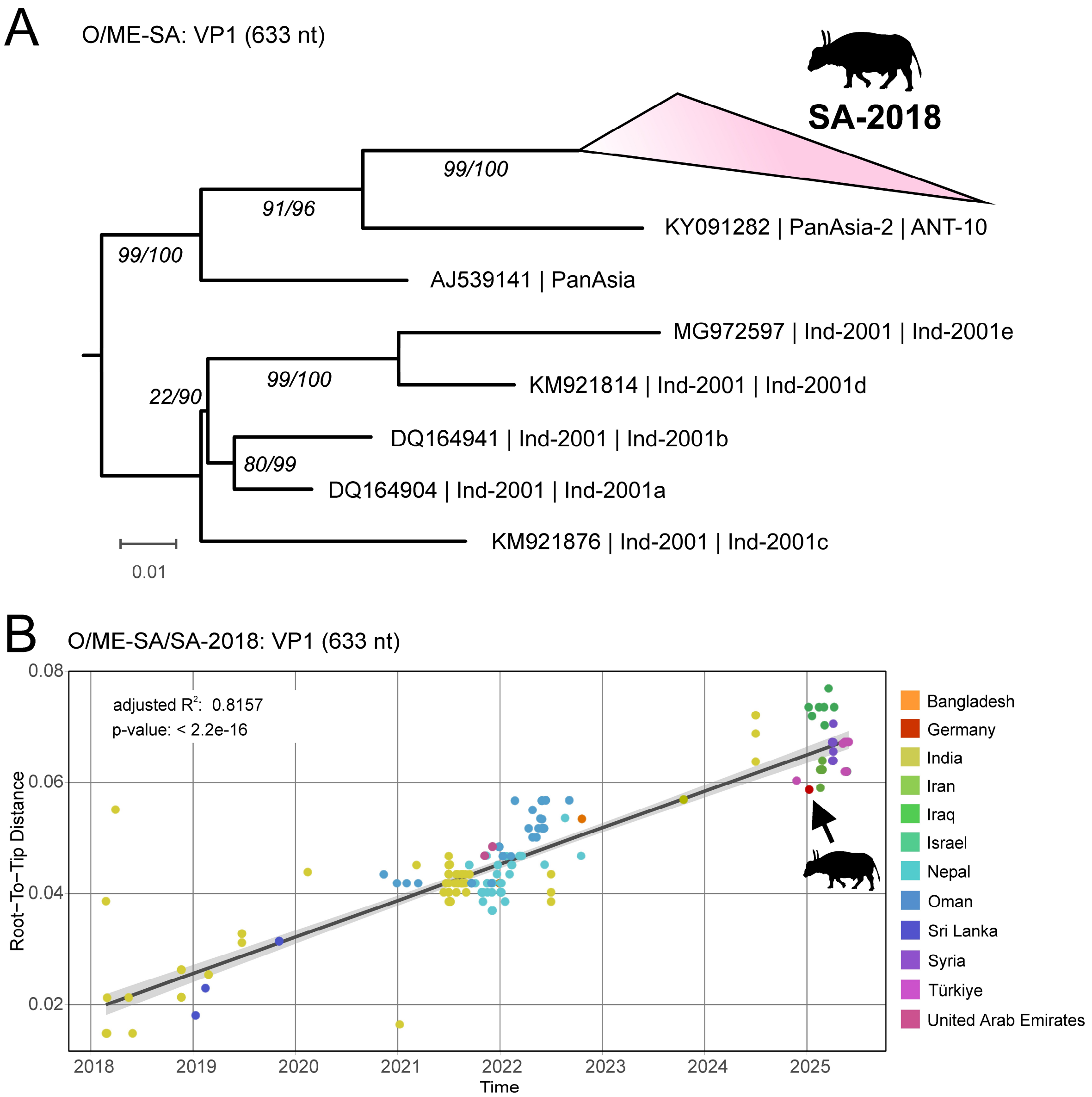

Supplemental Figure S2: Phylogenetic and temporal analysis of the FMDV VP1-coding region. A maximum-likelihood phylogenetic tree showing the German FMDV VP1 sequence alongside 194 reference sequences from lineage SA-2018 and 7 representative sequences from lineages Ind-2001, PanAsia and PanAsia-2, all within topotype O/ME-SA (A). The tree was

inferred using IQ-TREE (v3.0.1) with automatic model selection and branch support estimated from each 10,000 ultrafast bootstrap and SH-aLRT replicates. Branch support is shown in italics using the scheme: SH-aLRT support (%) / ultrafast bootstrap support (%). The correlation of genetic divergence (root-to-tip distance) with the sampling date for sequences from lineage SA-2018 (n = 195) was estimated using TempEst (v1.5.3) (B). The linear regression line and coefficient of determination (R^2) were calculated in R (v4.3.1) using the 'lm' function.

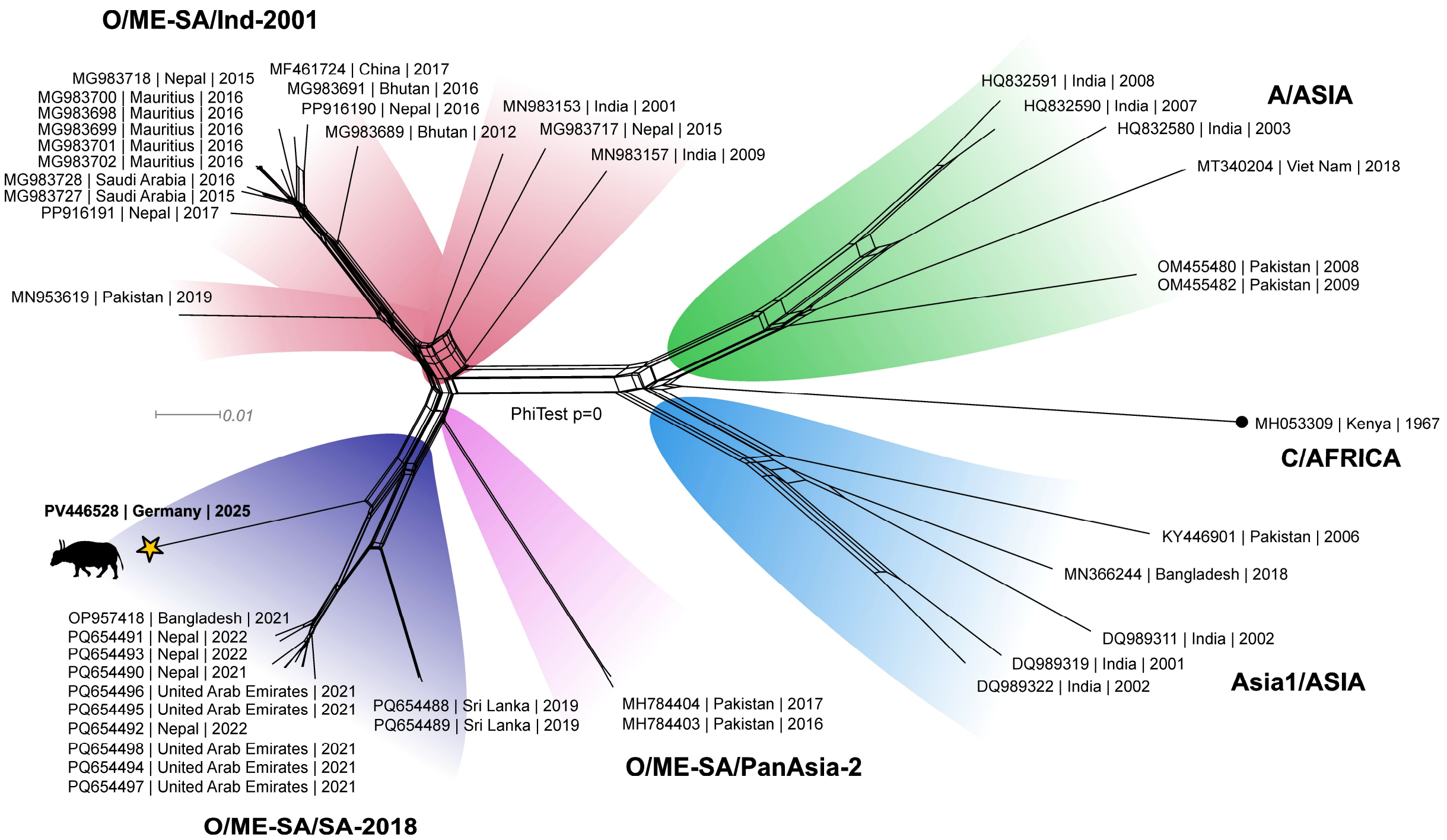

Supplemental Figure S3: Phylogenetic network of full FMDV genome sequences shows evidence of recombination.

NeighborNet analysis using an alignment of 43 selected full FMDV genome reference sequences from different lineages along with the German FMDV strain. Phylogenetic conflicting signals, e.g., caused by recombination events, are visualized as box-like structures in the net. The German FMDV strain (star symbol) groups with members of the SA-2018 lineage but its offset position in relation to the other strains indicates recombination. A PhiTest showed statistically significant support for recombination (p-value=0).

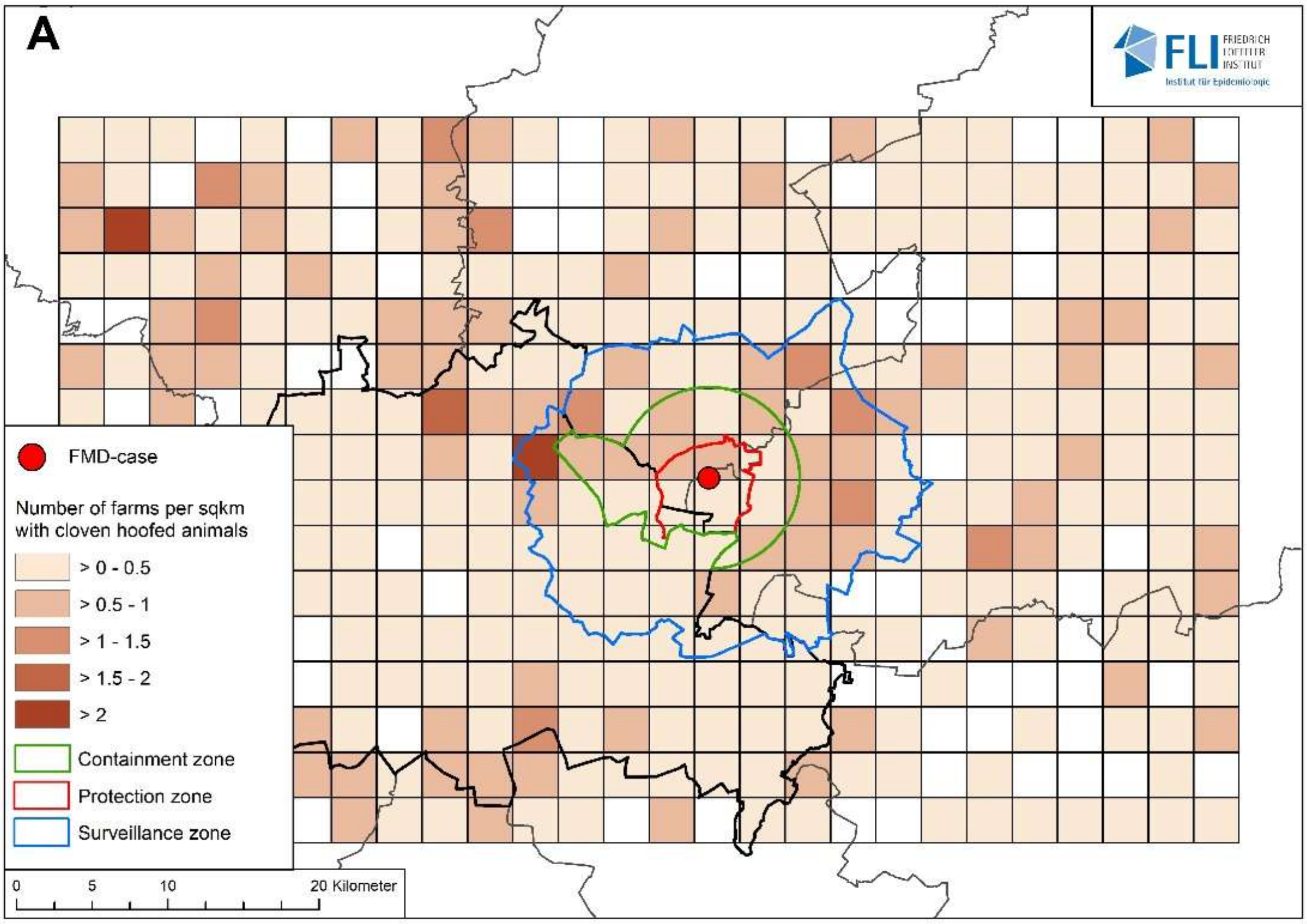
A
FLI FRIEDRICH LOEFFLER INSTITUT
Institut für Epidemiologie
FMD-case
Number of farms per sqkm
with cloven hoofed animals
> 0 - 0.5
> 0.5 - 1
> 1 - 1.5
> 1.5 - 2
> 2
Containment zone
Protection zone
Surveillance zone
0
5
10
20 Kilometer

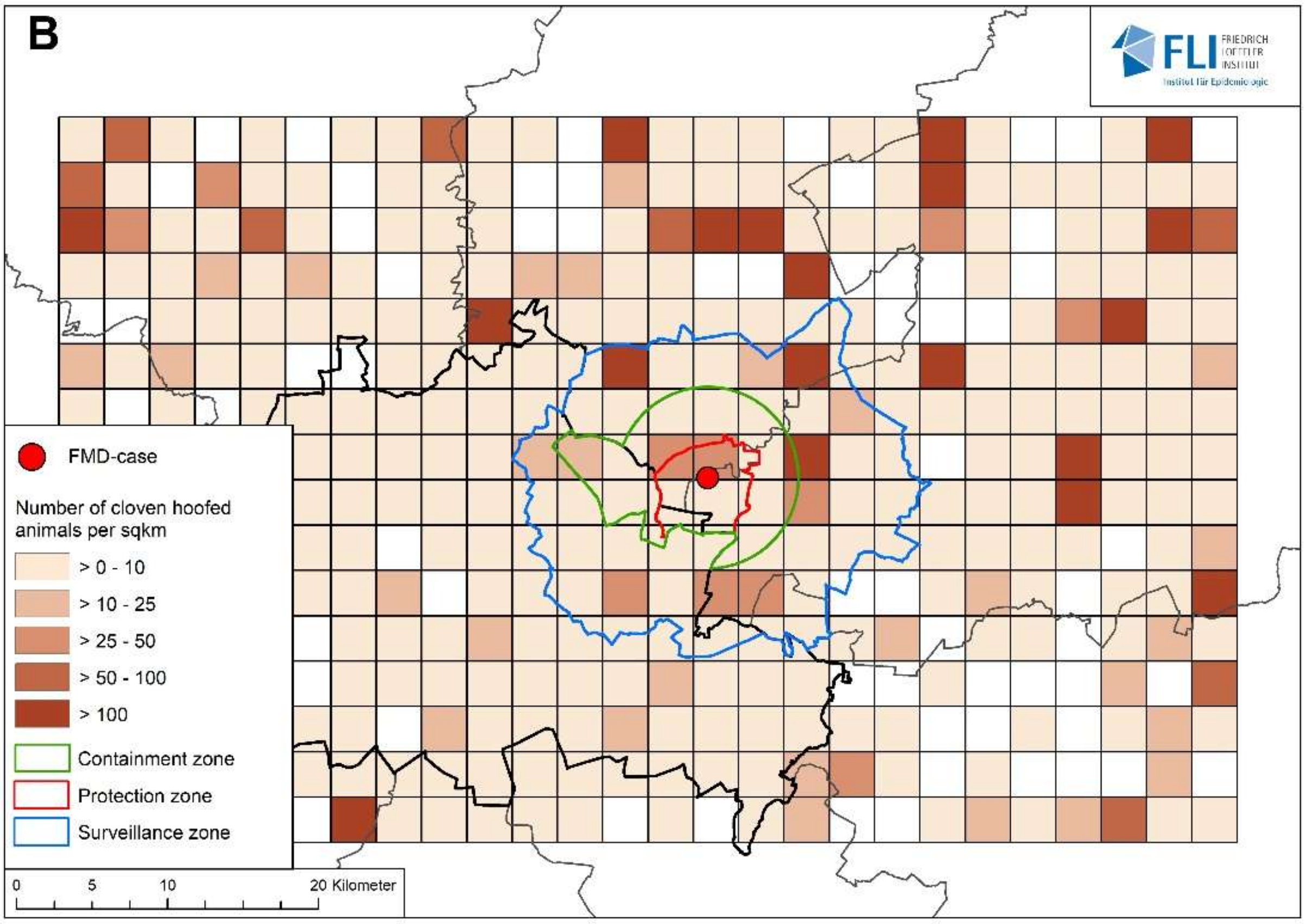
B
FLI FRIEDRICH LOEFFLER INSTITUT
Institut für Epidemiologie
FMD-case
Number of cloven hoofed
animals per sqkm
> 0 - 10
> 10 - 25
> 25 - 50
> 50 - 100
> 100
Containment zone
Protection zone
Surveillance zone
0
5
10
20 Kilometer

Supplemental Figure S4: Farm and livestock density. Density of farms with cloven-hoofed animals (A) and density of cloven-hoofed animals (B) around the outbreak farm. For comparison, the median density of cloven-hoofed livestock in Germany is 42 per km^2 (Q1–Q3: 19–85). The red and blue lines show the protection and surveillance zone declared around the outbreak farm on 10 January 2025. The green line shows the containment zone, which was put in place on 12 March 2025.

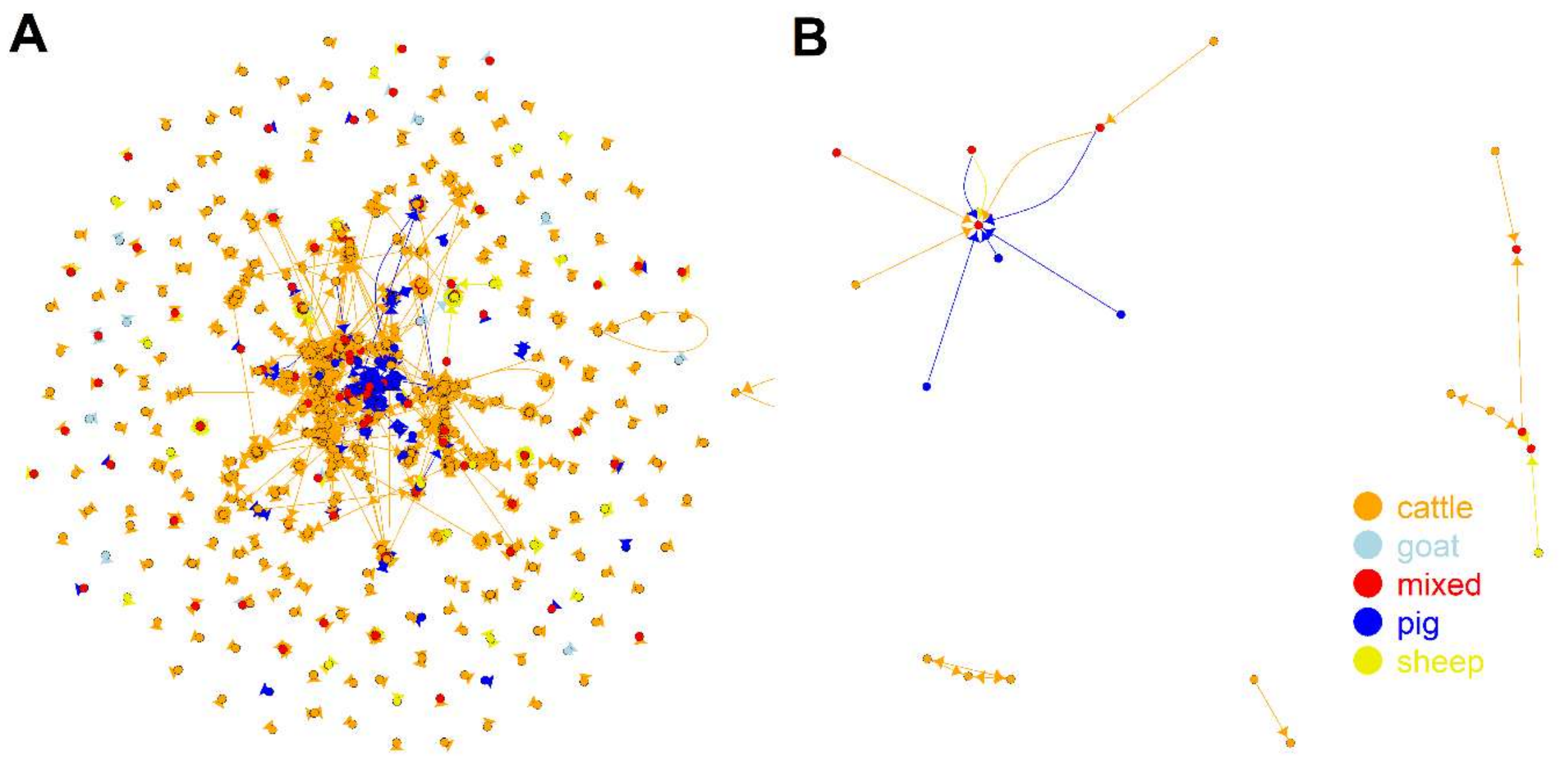

Supplemental Figure S5: Trade network of animals. Cattle, swine, goat and sheep movements in (A) Berlin and Brandenburg and (B) the restricted zones from 1 December 2024 to 9 January 2025. Node and edge colours indicate the species of the animals that were transported.

Supplemental Tables

Supplemental Table S1: Virological and serological results for 11 culled water buffalo

Results for the animal (ear tag 334) that had died on the farm on 5 January 2025 and was submitted for necropsy at the state veterinary diagnostic laboratory are not included in this table. No swab samples or lesion material were collected from this animal. The cut-off for the antibody ELISAs is at S/N% 50.

**animal** *(ear tag)*	**serum** *(FMDV Cq)*	**nasal swab** *(FMDV Cq)*	**mouth lesion** *(FMDV Cq)*	**foot lesion** *(FMDV Cq)*	**oldest lesion** *(age in days)*	**NSP antibody ELISA** *(S/N%)*	**Type O SP antibody ELISA** *(S/N%)*
168	33.5	32.8	none found	22.6	not determined	pos (13)	pos (22)
739	39.7	32.4	32.7	20.9	5	pos (18)	pos (35)
165	neg (No Cq)	37.1	none found	none found	not determined	pos (31)	pos (22)
565	neg (No Cq)	35.8	34.0	none found	>7	pos (24)	pos (19)
779	neg (No Cq)	33.8	35.5	none found	4-5	pos (13)	pos (19)
170	neg (No Cq)	neg	39.0	none found	5	pos (29)	pos (13)
465	35.7	37.7	36.1	none found	5	pos (27)	pos (22)
740	neg (No Cq)	32.4	37.8	none found	not determined	pos (16)	pos (29)
169	37.8	31.3	neg (No Cq)	none found	3	pos (35)	pos (28)
113	neg (No Cq)	33.9	neg (No Cq)	none found	not determined	pos (19)	pos (26)
167	neg (No Cq)	35.2	neg (No Cq)	none found	not determined	pos (40)	pos (37)

Supplemental Table S2: Overview of the available samples and those selected for library preparation and sequencing. The samples/libraries that were sequenced are underlined in the table. Samples that were not sequenced are indicated in grey. The samples were selected based on the criteria defined by Ebinger *et al.* 2021, the quantification results obtained for the respective libraries (library [pM]), and the results of quality control in order to maximize the likelihood of success.

Sample			Implen RNA quantification and quality control								Sample rating			Sequencing libraries		
ID (250110-07135767-)	Material	RT-qPCR 3D-OIE	Concentration [ng/µl]	A230 (10 mm)	A260 (10 mm)	A280 (10 mm)	λ	Abs.	A260/A280	A260/A230	relCq	fmdvRel	fmdvRel/ Concentration	ONT	Ion Torrent	Ion Torrent Library Molarity [pM]
01	Lung	25.3	27.76	0.328	0.698	0.342	320	0.004	2.053	2.142	0.0	100.000	3.602E+00	lib07010	lib07003	23.1
06	Blood	33.4	3.56	0.182	0.096	0.060	320	0.007	1.679	0.509	8.1	0.364	1.024E-01	ND	lib07008	9.0
07	Lung	27.1	756.16	8.410	18.950	8.789	320	0.046	2.162	2.260	1.8	28.717	3.798E-02	lib07014	lib07009	110.3
02	Intestine	31.3	57.44	0.667	1.441	0.685	320	0.005	2.112	2.169	6.0	1.563	2.720E-02	lib07011	lib07004	50.5
04	Lymph node	30.5	106.44	1.354	2.662	1.259	320	0.001	2.115	1.967	5.2	2.720	2.556E-02	lib07012	lib07006	58.1
05	Tonsil	32.5	110.36	1.393	2.767	1.322	320	0.008	2.100	1.992	7.2	0.680	6.163E-03	lib07013	lib07007	99.8
08	Intestine	34.3	596.84	6.652	14.960	6.935	320	0.039	2.164	2.256	9.0	0.195	3.272E-04	ND	ND	NA
10	Lymph node	32.0	4990.8	55.810	125.200	57.680	320	0.430	2.179	2.253	6.7	0.962	1.927E-04	ND	ND	NA
03	Spleen	36.1	675.72	7.856	17.000	8.014	320	0.107	2.136	2.180	10.8	0.056	8.301E-05	ND	lib07005	48.7
11	Tonsil	35.4	1367.9	15.160	34.340	16.240	320	0.143	2.124	2.277	10.1	0.091	6.661E-05	ND	ND	NA
09	Spleen	37.3	2370.7	26.170	59.430	27.860	320	0.162	2.140	2.279	12.0	0.024	1.030E-05	ND	ND	NA

Supplemental Table S3: Summary of sequencing results

Sample		ONT sequencing						Ion Torrent sequencing					
ID	Material	Library ID	Reads total*	Reads FMDV	% FMDV reads	% Coverage (relative to OP957418.1)	Mean Depth of Coverage	Library ID	Reads total	Reads FMDV	% FMDV reads	% Coverage (relative to OP957418.1)	Median Depth of Coverage
250110-07135767-01	Lung	lib07010	512,971	2,532	0.494	100.0	108	lib07003	9,352,173	46,145	0.493	100.0	1824
250110-07135767-02	Intestine	lib07011	259,079	34	<0.001	68.0	1.6	lib07004	ND	ND	ND	ND	ND
250110-07135767-03	Spleen	ND	ND	ND	ND			lib07005	ND	ND	ND	ND	ND
250110-07135767-04	Lymph node	lib07012	282,437	1	<0.001	2.3	-	lib07006	ND	ND	ND	ND	ND
250110-07135767-05	Tonsil	lib07013	710,377	1	<0.001	2.9	-	lib07007	ND	ND	ND	ND	ND
250110-07135767-06	Blood	ND	ND	ND	ND			lib07008	6,649,486	313	0.005	98.2	12
250110-07135767-07	Lung	lib07014	771,833	206	0.026	96.2	9.7	lib07009	15,642,638	6,964	0.045	100.0	288
250110-07135767-08	Intestine	ND	ND	ND	ND			ND	ND	ND	ND	ND	ND
250110-07135767-09	Spleen	ND	ND	ND	ND			ND	ND	ND	ND	ND	ND
250110-07135767-10	Lymph node	ND	ND	ND	ND			ND	ND	ND	ND	ND	ND
250110-07135767-11	Tonsil	ND	ND	ND	ND			ND	ND	ND	ND	ND	ND

ND – not done. *Read numbers after quality trimming with BBDuk (v38.84) applying the following parameters: qtrim=rl trimq=10 minlength=100 ordered=t qin=33

Supplemental Table S4: Vaccine matching results

The r_1 values shown below represent the one-way serological matches between the vaccine strain and the field isolate, calculated from the comparative reactivity of a pool of bovine sera raised against the vaccine in question, to the vaccine virus and the field isolate. Heterologous neutralisation titres for the field isolate are provided. The result represents the mean of three independent repetitions.

Vaccine strain	Heterologous titre	r_1 value
O-3039 BI	2.00	0.28
O-PanAsia 2 BI	2.25	0.35
O_1 Manisa BI	2.15	0.63

Supplemental Table S5: Trading activity

Farm types and species traded to or from farms in Berlin and Brandenburg between 1 December 2024 and 9 January 2025, as reported to the German animal traceability and information system HI-Tier.

Farm type	Species traded	Transports	Animals
cattle	cattle	1,562	32,211
mixed	cattle	145	2,466
goat	goat	13	77
mixed	goat	17	277
mixed	pig	57	15,550
pig	pig	207	142,307
mixed	sheep	34	3,063
sheep	sheep	103	5,880
**Total:**		**2,138**	**201,831**

Supplemental Table S6: List of publicly available sequences used in this study

GenBank Accession	Analysis 1	Analysis 2	Analysis 3
PX738467	Phylogenetic analyses	Time-aware phylogenetic analyses	
PX704477	Phylogenetic analyses	Time-aware phylogenetic analyses	
PX704476	Phylogenetic analyses	Time-aware phylogenetic analyses	
PX704475	Phylogenetic analyses	Time-aware phylogenetic analyses	
PX704474	Phylogenetic analyses	Time-aware phylogenetic analyses	
PX704473	Phylogenetic analyses	Time-aware phylogenetic analyses	
PX704472	Phylogenetic analyses	Time-aware phylogenetic analyses	
PX704471	Phylogenetic analyses	Time-aware phylogenetic analyses	
PX698932	Phylogenetic analyses	Time-aware phylogenetic analyses	
PX698930	Phylogenetic analyses	Time-aware phylogenetic analyses	
PX698929	Phylogenetic analyses	Time-aware phylogenetic analyses	
PX698928	Phylogenetic analyses	Time-aware phylogenetic analyses	
PX698927	Phylogenetic analyses	Time-aware phylogenetic analyses	
PX698926	Phylogenetic analyses	Time-aware phylogenetic analyses	
PX685521	Phylogenetic analyses	Time-aware phylogenetic analyses	
PX685520	Phylogenetic analyses	Time-aware phylogenetic analyses	
PX685519	Phylogenetic analyses	Time-aware phylogenetic analyses	
PX685518	Phylogenetic analyses	Time-aware phylogenetic analyses	
PX685517	Phylogenetic analyses	Time-aware phylogenetic analyses	
PX685516	Phylogenetic analyses	Time-aware phylogenetic analyses	
PX685515	Phylogenetic analyses	Time-aware phylogenetic analyses	
PX685514	Phylogenetic analyses	Time-aware phylogenetic analyses	
PX685513	Phylogenetic analyses	Time-aware phylogenetic analyses	
PX685512	Phylogenetic analyses	Time-aware phylogenetic analyses	
PX685511	Phylogenetic analyses	Time-aware phylogenetic analyses	
PX685510	Phylogenetic analyses	Time-aware phylogenetic analyses	
PX685509	Phylogenetic analyses	Time-aware phylogenetic analyses	
PX685508	Phylogenetic analyses	Time-aware phylogenetic analyses	
PX685507	Phylogenetic analyses	Time-aware phylogenetic analyses	
PV446528	Phylogenetic analyses	Time-aware phylogenetic analyses	Recombination analysis
PV068830	Phylogenetic analyses	Time-aware phylogenetic analyses	
PV068829	Phylogenetic analyses	Time-aware phylogenetic analyses	
PV068828	Phylogenetic analyses	Time-aware phylogenetic analyses	
PV068827	Phylogenetic analyses	Time-aware phylogenetic analyses	
PQ654487	Phylogenetic analyses	Time-aware phylogenetic analyses	
PQ654486	Phylogenetic analyses	Time-aware phylogenetic analyses	
PQ654485	Phylogenetic analyses	Time-aware phylogenetic analyses	
PQ654484	Phylogenetic analyses	Time-aware phylogenetic analyses	
PQ654483	Phylogenetic analyses	Time-aware phylogenetic analyses	
PQ654482	Phylogenetic analyses	Time-aware phylogenetic analyses	
PQ654481	Phylogenetic analyses	Time-aware phylogenetic analyses	

PQ654480	Phylogenetic analyses	Time-aware phylogenetic analyses	
PQ654479	Phylogenetic analyses	Time-aware phylogenetic analyses	
PQ654478	Phylogenetic analyses	Time-aware phylogenetic analyses	
PQ654477	Phylogenetic analyses	Time-aware phylogenetic analyses	
PQ654476	Phylogenetic analyses	Time-aware phylogenetic analyses	
PQ654475	Phylogenetic analyses	Time-aware phylogenetic analyses	
PQ654474	Phylogenetic analyses	Time-aware phylogenetic analyses	
PQ654473	Phylogenetic analyses	Time-aware phylogenetic analyses	
PQ654472	Phylogenetic analyses	Time-aware phylogenetic analyses	
PQ654471	Phylogenetic analyses	Time-aware phylogenetic analyses	
PQ654470	Phylogenetic analyses	Time-aware phylogenetic analyses	
PQ654469	Phylogenetic analyses	Time-aware phylogenetic analyses	
PQ654468	Phylogenetic analyses	Time-aware phylogenetic analyses	
PQ654467	Phylogenetic analyses	Time-aware phylogenetic analyses	
PQ654466	Phylogenetic analyses	Time-aware phylogenetic analyses	
PQ654465	Phylogenetic analyses	Time-aware phylogenetic analyses	
PQ654464	Phylogenetic analyses	Time-aware phylogenetic analyses	
PQ654463	Phylogenetic analyses	Time-aware phylogenetic analyses	
PQ654462	Phylogenetic analyses	Time-aware phylogenetic analyses	
PQ654461	Phylogenetic analyses	Time-aware phylogenetic analyses	
PQ654460	Phylogenetic analyses	Time-aware phylogenetic analyses	
PQ654459	Phylogenetic analyses	Time-aware phylogenetic analyses	
PQ654458	Phylogenetic analyses	Time-aware phylogenetic analyses	
PQ654457	Phylogenetic analyses	Time-aware phylogenetic analyses	
PQ654456	Phylogenetic analyses	Time-aware phylogenetic analyses	
PQ654455	Phylogenetic analyses	Time-aware phylogenetic analyses	
PQ654454	Phylogenetic analyses	Time-aware phylogenetic analyses	
PQ654453	Phylogenetic analyses	Time-aware phylogenetic analyses	
PQ654452	Phylogenetic analyses	Time-aware phylogenetic analyses	
PQ654451	Phylogenetic analyses	Time-aware phylogenetic analyses	
PQ654450	Phylogenetic analyses	Time-aware phylogenetic analyses	
PQ654449	Phylogenetic analyses	Time-aware phylogenetic analyses	
PQ654448	Phylogenetic analyses	Time-aware phylogenetic analyses	
PQ654447	Phylogenetic analyses	Time-aware phylogenetic analyses	
PQ654446	Phylogenetic analyses	Time-aware phylogenetic analyses	
PQ654445	Phylogenetic analyses	Time-aware phylogenetic analyses	
OR991332	Phylogenetic analyses	Time-aware phylogenetic analyses	
OR934914	Phylogenetic analyses	Time-aware phylogenetic analyses	
OR934913	Phylogenetic analyses	Time-aware phylogenetic analyses	
OR934912	Phylogenetic analyses	Time-aware phylogenetic analyses	
OR934911	Phylogenetic analyses	Time-aware phylogenetic analyses	
OR934910	Phylogenetic analyses	Time-aware phylogenetic analyses	
OR934909	Phylogenetic analyses	Time-aware phylogenetic analyses	
OR934908	Phylogenetic analyses	Time-aware phylogenetic analyses	
OR934907	Phylogenetic analyses	Time-aware phylogenetic analyses	
OR934906	Phylogenetic analyses	Time-aware phylogenetic analyses	
OR934905	Phylogenetic analyses	Time-aware phylogenetic analyses	

OR934904	Phylogenetic analyses	Time-aware phylogenetic analyses	
OR934903	Phylogenetic analyses	Time-aware phylogenetic analyses	
OR934902	Phylogenetic analyses	Time-aware phylogenetic analyses	
OR934901	Phylogenetic analyses	Time-aware phylogenetic analyses	
OR934900	Phylogenetic analyses	Time-aware phylogenetic analyses	
OR934899	Phylogenetic analyses	Time-aware phylogenetic analyses	
OR934897	Phylogenetic analyses	Time-aware phylogenetic analyses	
OR934893	Phylogenetic analyses	Time-aware phylogenetic analyses	
OR934892	Phylogenetic analyses	Time-aware phylogenetic analyses	
OR934884	Phylogenetic analyses	Time-aware phylogenetic analyses	
OR934883	Phylogenetic analyses	Time-aware phylogenetic analyses	
OR934882	Phylogenetic analyses	Time-aware phylogenetic analyses	
OR934881	Phylogenetic analyses	Time-aware phylogenetic analyses	
OR934880	Phylogenetic analyses	Time-aware phylogenetic analyses	
OR934878	Phylogenetic analyses	Time-aware phylogenetic analyses	
OR934877	Phylogenetic analyses	Time-aware phylogenetic analyses	
OR934876	Phylogenetic analyses	Time-aware phylogenetic analyses	
OR934875	Phylogenetic analyses	Time-aware phylogenetic analyses	
OR934874	Phylogenetic analyses	Time-aware phylogenetic analyses	
OR934873	Phylogenetic analyses	Time-aware phylogenetic analyses	
OR934872	Phylogenetic analyses	Time-aware phylogenetic analyses	
OR934871	Phylogenetic analyses	Time-aware phylogenetic analyses	
OR934870	Phylogenetic analyses	Time-aware phylogenetic analyses	
OR934868	Phylogenetic analyses	Time-aware phylogenetic analyses	
OR934863	Phylogenetic analyses	Time-aware phylogenetic analyses	
OR934862	Phylogenetic analyses	Time-aware phylogenetic analyses	
OR934861	Phylogenetic analyses	Time-aware phylogenetic analyses	
OR425057	Phylogenetic analyses	Time-aware phylogenetic analyses	
OR425056	Phylogenetic analyses	Time-aware phylogenetic analyses	
OR425055	Phylogenetic analyses	Time-aware phylogenetic analyses	
OR425054	Phylogenetic analyses	Time-aware phylogenetic analyses	
OR425053	Phylogenetic analyses	Time-aware phylogenetic analyses	
OQ847053	Phylogenetic analyses	Time-aware phylogenetic analyses	
OQ847052	Phylogenetic analyses	Time-aware phylogenetic analyses	
OQ847051	Phylogenetic analyses	Time-aware phylogenetic analyses	
OQ847050	Phylogenetic analyses	Time-aware phylogenetic analyses	
OQ847049	Phylogenetic analyses	Time-aware phylogenetic analyses	
OQ847048	Phylogenetic analyses	Time-aware phylogenetic analyses	
OQ847047	Phylogenetic analyses	Time-aware phylogenetic analyses	
OQ847046	Phylogenetic analyses	Time-aware phylogenetic analyses	
OQ847044	Phylogenetic analyses	Time-aware phylogenetic analyses	
OQ847043	Phylogenetic analyses	Time-aware phylogenetic analyses	
OQ847042	Phylogenetic analyses	Time-aware phylogenetic analyses	
OQ847041	Phylogenetic analyses	Time-aware phylogenetic analyses	
OQ847030	Phylogenetic analyses	Time-aware phylogenetic analyses	
OQ847029	Phylogenetic analyses	Time-aware phylogenetic analyses	
OQ847027	Phylogenetic analyses	Time-aware phylogenetic analyses	

OQ847026	Phylogenetic analyses	Time-aware phylogenetic analyses	
OQ847025	Phylogenetic analyses	Time-aware phylogenetic analyses	
OQ847024	Phylogenetic analyses	Time-aware phylogenetic analyses	
OQ847023	Phylogenetic analyses	Time-aware phylogenetic analyses	
OQ847022	Phylogenetic analyses	Time-aware phylogenetic analyses	
OQ847021	Phylogenetic analyses	Time-aware phylogenetic analyses	
OQ847019	Phylogenetic analyses	Time-aware phylogenetic analyses	
OQ847018	Phylogenetic analyses	Time-aware phylogenetic analyses	
OQ847017	Phylogenetic analyses	Time-aware phylogenetic analyses	
OQ847016	Phylogenetic analyses	Time-aware phylogenetic analyses	
OQ847015	Phylogenetic analyses	Time-aware phylogenetic analyses	
OQ847014	Phylogenetic analyses	Time-aware phylogenetic analyses	
OQ847013	Phylogenetic analyses	Time-aware phylogenetic analyses	
OQ847012	Phylogenetic analyses	Time-aware phylogenetic analyses	
OQ847011	Phylogenetic analyses	Time-aware phylogenetic analyses	
OQ847010	Phylogenetic analyses	Time-aware phylogenetic analyses	
OQ847007	Phylogenetic analyses	Time-aware phylogenetic analyses	
OQ847006	Phylogenetic analyses	Time-aware phylogenetic analyses	
OQ847005	Phylogenetic analyses	Time-aware phylogenetic analyses	
OQ847004	Phylogenetic analyses	Time-aware phylogenetic analyses	
OQ846965	Phylogenetic analyses	Time-aware phylogenetic analyses	
OQ846964	Phylogenetic analyses	Time-aware phylogenetic analyses	
OQ846963	Phylogenetic analyses	Time-aware phylogenetic analyses	
OQ846962	Phylogenetic analyses	Time-aware phylogenetic analyses	
OQ846961	Phylogenetic analyses	Time-aware phylogenetic analyses	
OQ846960	Phylogenetic analyses	Time-aware phylogenetic analyses	
OQ846959	Phylogenetic analyses	Time-aware phylogenetic analyses	
OQ846958	Phylogenetic analyses	Time-aware phylogenetic analyses	
OQ846957	Phylogenetic analyses	Time-aware phylogenetic analyses	
OQ846956	Phylogenetic analyses	Time-aware phylogenetic analyses	
OQ846955	Phylogenetic analyses	Time-aware phylogenetic analyses	
OQ846954	Phylogenetic analyses	Time-aware phylogenetic analyses	
OQ846953	Phylogenetic analyses	Time-aware phylogenetic analyses	
OQ806933	Phylogenetic analyses	Time-aware phylogenetic analyses	
OQ732592	Phylogenetic analyses	Time-aware phylogenetic analyses	
OQ732591	Phylogenetic analyses	Time-aware phylogenetic analyses	
OQ732590	Phylogenetic analyses	Time-aware phylogenetic analyses	
OQ732589	Phylogenetic analyses	Time-aware phylogenetic analyses	
OQ732588	Phylogenetic analyses	Time-aware phylogenetic analyses	
OQ732576	Phylogenetic analyses	Time-aware phylogenetic analyses	
OQ732575	Phylogenetic analyses	Time-aware phylogenetic analyses	
OP913189	Phylogenetic analyses	Time-aware phylogenetic analyses	
OP320458	Phylogenetic analyses	Time-aware phylogenetic analyses	
OP320457	Phylogenetic analyses	Time-aware phylogenetic analyses	
OP320456	Phylogenetic analyses	Time-aware phylogenetic analyses	
OP320455	Phylogenetic analyses	Time-aware phylogenetic analyses	
MT936116	Phylogenetic analyses	Time-aware phylogenetic analyses	

MT936115	Phylogenetic analyses	Time-aware phylogenetic analyses	
MT936114	Phylogenetic analyses	Time-aware phylogenetic analyses	
MT936113	Phylogenetic analyses	Time-aware phylogenetic analyses	
MT936111	Phylogenetic analyses	Time-aware phylogenetic analyses	
MT936110	Phylogenetic analyses	Time-aware phylogenetic analyses	
MT919005	Phylogenetic analyses	Time-aware phylogenetic analyses	
MT919004	Phylogenetic analyses	Time-aware phylogenetic analyses	
MT919002	Phylogenetic analyses	Time-aware phylogenetic analyses	
MT918999	Phylogenetic analyses	Time-aware phylogenetic analyses	
MT918997	Phylogenetic analyses	Time-aware phylogenetic analyses	
MT918995	Phylogenetic analyses	Time-aware phylogenetic analyses	
MT918994	Phylogenetic analyses	Time-aware phylogenetic analyses	
MG972597	Phylogenetic analyses		
KY091282	Phylogenetic analyses		
KM921876	Phylogenetic analyses		
KM921814	Phylogenetic analyses		
DQ164941	Phylogenetic analyses		
DQ164904	Phylogenetic analyses		
AJ539141	Phylogenetic analyses		
PQ654498			Recombination analysis
PQ654497			Recombination analysis
PQ654496			Recombination analysis
PQ654495			Recombination analysis
PQ654494			Recombination analysis
PQ654493			Recombination analysis
PQ654492			Recombination analysis
PQ654491			Recombination analysis
PQ654490			Recombination analysis
PQ654489			Recombination analysis
PQ654488			Recombination analysis
PP916191			Recombination analysis
PP916190			Recombination analysis
OP957418			Recombination analysis
OM455482			Recombination analysis
OM455480			Recombination analysis
MT340204			Recombination analysis
MN983157			Recombination analysis
MN983153			Recombination analysis
MN953619			Recombination analysis
MN366244			Recombination analysis
MH784404			Recombination analysis
MH784403			Recombination analysis
MH053309			Recombination analysis
MG983728			Recombination analysis
MG983727			Recombination analysis
MG983718			Recombination analysis
MG983717			Recombination analysis

MG983702			Recombination analysis
MG983701			Recombination analysis
MG983700			Recombination analysis
MG983699			Recombination analysis
MG983698			Recombination analysis
MG983691			Recombination analysis
MG983689			Recombination analysis
MF461724			Recombination analysis
KY446901			Recombination analysis
HQ832591			Recombination analysis
HQ832590			Recombination analysis
HQ832580			Recombination analysis
DQ989322			Recombination analysis
DQ989319			Recombination analysis
DQ989311			Recombination analysis
